# Using AAEHS-Net as an Attention-Based Auxiliary Extraction and Hybrid Subsampled Network for Semantic Segmentation

**DOI:** 10.1155/2022/1536976

**Published:** 2022-10-14

**Authors:** Shan Zhao, Yibo Wang, Kaiwen Tian

**Affiliations:** School of Software, Henan Polytechnic University, Jiaozuo 454003, China

## Abstract

Semantic segmentation based on deep learning has undergone remarkable advancements in recent years. However, due to the neglect of the shallow features, the problems of inaccurate segmentation have persisted. To address this issue, a semantic segmentation network-attention-based auxiliary extraction and hybrid subsampled network (AAEHS-Net) is suggested in this study. To extract more deep information and the shallow features, the complementary and enhanced extraction module (CEEM) is utilized by the network. As a result, the edge segmentation of the model is improved. Moreover, to reduce the loss of features, a hybrid subsampled module (HSM) is introduced. Meanwhile, global max pool and global avg pool module (GAGM) is designed as an attention module to enhance the features with global and important information and maintain feature continuity. The proposed AAEHS-Net is evaluated on three datasets: the aerial drone image dataset, the Massachusetts roads dataset, and the Massachusetts buildings dataset. On the three datasets, AAEHS-Net achieves 1.15%, 0.88%, and 2.1% higher accuracy than U-Net, reaching 90.12%, 96.23%, and 95.15%, respectively. At the same time, our proposed network has obtained the best values for all evaluation metrics in three datasets compared to the currently popular algorithms.

## 1. Introduction

As a basic assignment in computer vision research, semantic segmentation allocates a class label to each pixel of the picture. However, this is a challenging assignment [1]. Moreover, due to providing category information at the pixel level, semantic segmentation plays a key role in numerous applications and areas [2], such as self-driving vehicles [3, 4], medical image analysis [5], image classification [6], target detection [7], and face recognition [8, 9]. Over the last decades, numerous techniques have been proposed for image segmentation. In general, segmentation methods can be broadly classified into two categories of traditional methods and deep neural network-based methods.

While in the former methods, such basic attributes of the image as the regions [10], edges [11], and thresholds [12] are used to achieve segmentation. In this case, the region-based methods extract the region by considering the similar characteristics of the pixels in terms of spatial and grey information. Nevertheless, the challenge lies in the initial division and the setting of the split-merge similarity measurement. While it is suitable for complex images, the high computational complexity as well as the destruction of the boundaries when splitting can be mentioned as the drawbacks. The edge-based segmentation methods detect the edge pixels at the boundaries and connect them to form edge contours, thus the image is divided into different regions. However, while these methods enjoy a simple computational process and a relatively effective performance, the algorithms are susceptible to noise. Yet, threshold-based methods adopt one or more thresholds and compare them to every pixel of the picture. Based on the comparison results, the pixels are classified into the appropriate category. These methods are simple and highly efficient, particularly for images with a uniform grey or less grey difference between the target and the background. However, while the grey of the pixels are taken into consideration, the semantic, spatial, and other feature information of the image are overlooked. Besides, the methods are susceptible to noise and, accordingly, not proper for complex images. So, to summarize, it can be argued that the traditional approaches rely heavily on manually designed features and suffer from certain drawbacks.

In recent years, the great development of neural networks has turned them into the dominant resolution for semantic segmentation. For example, to extract local features, AlexNet [6] adopts convolution, which highly improves the performance of segmentation over traditional methods. Fully convolutional neural networks (FCN) [13] enables end-to-end segmentation by classifying images at the pixel level, and accordingly, solving the problem of merely accepting fixed-size input images. In addition, inspired by the FCN, several convolutional neural networks are proposed. For example, to extract deeper features, the residual network (ResNet) [14] improves accuracy by adding to the depth of the network. As a result, the integrity of the information is protected through strengthening the initial features and mitigating the disappearance of the gradient. Moreover, to capture information at different scales, a pyramid pooling module is adopted by the pyramid scene parsing network (PSPNet) [15]. This leads to the aggregation of the contexts of different regions, which enables the model to understand the global information.

The typical encoder-decoder based network U-Net [16] shows a good segmentation performance for all variants of convolutional neural networks (CNN). It consists of an encoder and a decoder. Moreover, to connect the different features of the encoder and the decoder and obtain a more accurate segmentation, skip connections are added to the network which allows the spatial information to be applied directly to the deeper layers. With the popularity of U-Net, several innovative models have been introduced. Utilizing superimposition, U-Net++ [17] integrates the features of all stages in down sampling, the result of which is a fused feature map that contains numerous features. Swin-UNet [18] combines U-Net with a transformer [19] and replaces the original encoder and decoder completely with a transformer, with good results. However, the excessive parameters used in the network can be stated as its disadvantage. C-UNet [20] introduces two network models, namely, the original U-Net and a multiscale dense dilated convolution U-Net. In this network, the end result is a fusion of the prediction maps from the two networks. However, in contrast to the other methods, C-UNet requires a more complex training process. To extract features, SmaAt-UNet [21] uses attention modules and depthwise-separable convolutions, which results in a good segmentation with fewer parameters. Furthermore, proposed by Wang et al., ECA-Net [22] improves the channel attention, overcomes the paradox of performance and complexity trade-off, and reduces the complexity of the model. Nevertheless, spatial attention is not considered by the network. Moreover, as a new semantic segmentation network based on spatial and channel attention, SCAttNet [23] integrates lightweight spatial and channel attention modules and, consequently, adaptively refine the features.

Despite the achievement of rather good segmentation results, not only the above mentioned networks do not consider the shallow information, but an increase in the network layers leads to the less desirable segmentation of edge details. At the same time, since some level of accuracy is lost in the down-sampling process, the resulted precision loss leads to large semantic differences in the up-sampling process. Hence, the present study proposes an attention-based auxiliary extraction and hybrid subsampled network (AAEHS-Net) through which more abundant deep features are extracted by continuous multilayer residual convolutions. Moreover, the edge information is effectively utilized in the proposed model. In addition, the network captures more information and reduces feature loss, the important features are enhanced through fusion which in turn improves the segmentation accuracy. Furthermore, on the two datasets considered in this study, superior performance is obtained by the proposed network. Hence, the major contributions of the study are summarized as follows:

1. A complementary and enhanced extraction module (CEEM) is introduced to improve the edge feature extraction capability of the segmentation model. It consists of two branches, namely, the main branch and the auxiliary one, which extract deep and shallow features, respectively, and fuse them to enhance the effect on edge segmentation.
2. A hybrid subsampled module (HSM) is set forth, in which a convolutional combination is utilized, and an attention mechanism is introduced after convolution. Thus, the loss of features is improved effectively.
3. global max pool and global avg pool module (GAGM) is presented to enhance the integrity of the contextual information as well as to improve the performance of segmentation. Using GAGM, the global and shallow information are captured separately and are fused together later.
4. The comparison of the proposed model in this study with those of the U-Net family set forth in recent years revealed improved experimental results obtained by the present model.

The rest of this paper is organized as follows: Section 2 reviews the literature of the study. Next, the proposed AAEHS-Net is illustrated in Section 3. In Section 4, the experimental results and discussion are presented. Finally, the conclusions are given in Section 5.

## 2. Related Work

### 2.1. U-Net

**Figure 1:**
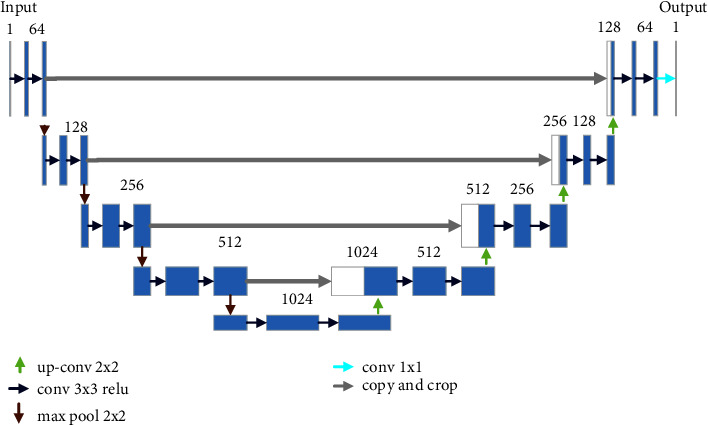
Overall architecture of the U-Net [16].

U-Net is an improved fully convolutional network model, the structure of which is displayed in Figure 1 [16]. It is a successful encoder-decoder network. The encoder is adopted for extracting features. While each module contains two convolutions with the size of 3 × 3, the number of channels is different, i.e., 64, 128, 256, 512, and 1024, respectively. The decoder consists of a deconvolution, a concatenation, and two convolutions. The number of channels is the exact opposite of that of the encoder. To compress the feature map, the pooling operation is introduced. As a result, it becomes smaller, and the complexity of the network is reduced. In contrast to pooling operations, deconvolution is intended to expand the features. The concatenation operation is to obtain more initial features.

The complete process of U-Net is as follows: each time encoding is passed, the size of the feature is halved, and the number of channels is doubled. This is done until the number of channels is 1024. Moreover, the decoding section corresponds to the encoding one. Then, to recover the corresponding feature size and the number of channels, the decoding process is carried out. Subsequently, it is stitched with the encoded features at the corresponding positions. To obtain the final output, the decoding operation is repeated until the feature size is restored to its original size. Afterwards, the number of channels is adjusted with the convolution, the sigmoid or softmax is activated, and the final segmented image is obtained.

### 2.2. Attention Mechanism

The performance of numerous models has been remarkably improved by attention mechanism in recent years. Therefore, the mechanism has been widely used in such fields as image segmentation, speech recognition [24], and machine translation [25].

**Figure 2:**
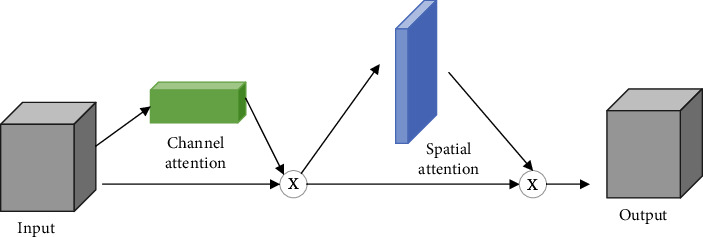
CBAM attention mechanism.

As a kind of dual attention mechanism, the convolutional block attention module (CBAM) [26] contains two independent submodules, namely, the channel attention module and the spatial attention module.  Figure 2 shows the structure of CBAM. Taking the feature map as the input, CBAM infers the attention map along the channel and spatial dimensions. Then, for the purpose of adaptive feature refinement, the attention map is sequentially multiplied by the input feature map, leading to the considerable improvement of its performance, while keeping the overhead small.

**Figure 3:**
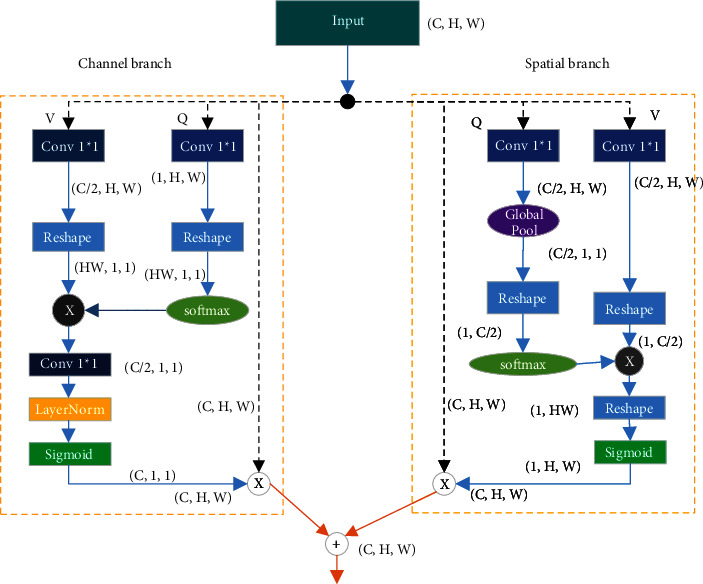
PSA attention mechanism.

The polarized self-attention (PSA) [27] (Figure 3) is divided into two branches, one for the channel dimension and the other for the spatial dimension. Regarding the former, the input feature is converted into the *Q* and *V* vectors with a convolution. Furthermore, the *Q* vector is augmented with softmax. Subsequently, matrix multiplication and convolution operations are performed to integrate the feature. Subsequently, LayerNorm is adopted to raise the dimension of the channel. Finally, a sigmoid function is used to achieve dynamic mapping.

## 3. AAEHS-Net

In this section, AAEHS-Net is introduced in details. First, the whole architecture of AAEHS-Net is illustrated in Section 3.1, and then, the structures of the three important components, i.e., the complementary and enhanced extraction module (CEEM), hybrid subsampled module (HSM), and GlobalMaxpool and GlobalAvgpool Module (GAGM) are provided in Section 3.2, Section 3.3, and Section 3.4, respectively. The algorithm flow is given in Section 3.5.

### 3.1. Overall Architecture

**Figure 4:**
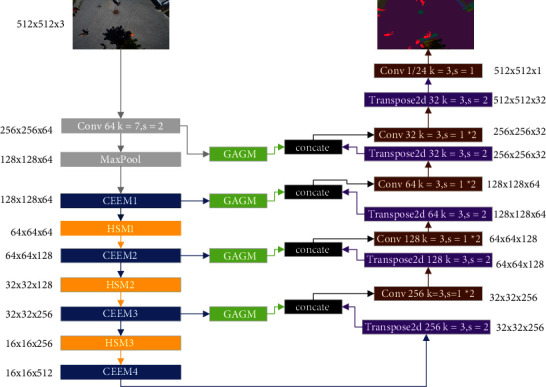
The architecture of our proposed AAEHS-Net.

Despite demonstrating effective results, U-Net-based segmentation networks suffer from two urgent questions. While the first issue is ignoring the shallow features, which leads to poor edge segmentation, the second is the degradation of precision due to the pooling operation. To solve these issues, a new semantic segmentation network (AAEHS-Net) is proposed. The overall architecture of the model is illustrated in Figure 4. As can be seen in the figure, AAEHS-Net is an end-to-end neural network.

Firstly, to extract more features as well as to quickly reduce the scale, the input image goes through a 7 × 7 convolution (strides = 2, channel = 64), followed by a max pooling operation. Subsequently, feature extraction is performed using CEEM. Moreover, for reducing the computational efforts, the features output from the CEEM are passed through the proposed HSM for feature compression. For ease of description, CEEM and HSM are collectively referred to as the encoding process. With each encoding process, the size of the feature map is halved, and the channel is doubled. Then, the encoding process is repeated for three times until the number of channels reaches 512. The next step is the decoding operation. Like U-Net, it contains a deconvolution, a skip connection, and two normal convolutions. While the purpose of the deconvolution is to restore the size of the feature map, the skip connection combines deep features with shallow features. Moreover, to maintain better contextual integrity of the combined features as well as to enhance shallow features, GAGM is introduced. The features obtained from the encoding process are first subjected to a deconvolution, while the skip connection features are enhanced by GAGM, subsequently fusing the two parts. The fused features are subjected to feature extraction again by twice convolution. This is a complete decoding process which needs to be repeated four times. Finally, to obtain the final output, the channel count is adjusted using convolution. While reducing feature loss from subsampling, AAEHS-Net improves the extraction of edge information. Using attention, the integrity of the contextual information is ensured by enhancing the features before fusion. In the next section, the details of the network and the design ideas are described.

### 3.2. CEEM (Complementary and Enhanced Extraction Module)

**Figure 5:**
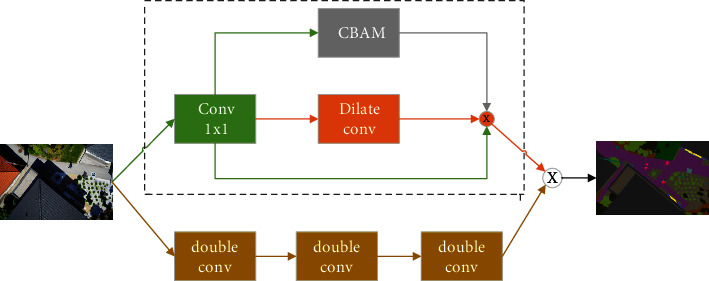
Proposed CEEM. The dashed box shows the ASM.

**Figure 6:**
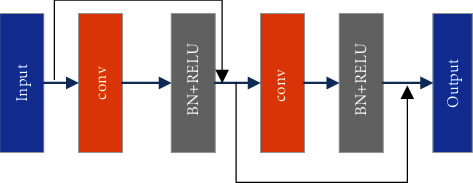
Architecture of the proposed double conv.

Image semantic segmentation is considered as a dense classification task, which focuses on the extraction of deep feature information. Moreover, network depth is a critical factor in determining the accuracy of segmentation [28]. The deeper the network is, the deeper features can be extracted. In order to extract the features, the traditional U-Net uses two stacked convolutions, which can easily cause inadequate extraction. To address this issue, the CEEM is designed. The specific structure of CEEM is shown in Figure 5. As can be seen in the figure, it has two parts, namely, the main structure and the auxiliary structure, which are referred to as MSM and ASM, respectively. ResNet34 is used as the backbone of the MSM. There are a number of reasons for the choice, including its numerous network layers for feature extraction, as well as its residual structure which prevents it from degrading the extraction power due to the high number of the layers. The MSM consists of multiple double convolutions, the structure of which is shown in Figure 6. As can be seen in the figure, it consists of two sets of convolutions and residuals. While the former is used for feature extraction, the latter fuses more initial features. Each convolution operation contains a normalization and a linear activation. Meanwhile, to reduce the computation complexity, the numbers of double convolutions in each MSM are changed to 3, 4, 5, and 2 in the present study. The specific information is indicated in Table 1. As can be seen in the table, CEEM1 contains both MSM1 and ADM1, with an input and an output size of 128 × 128 × 64. Although an increase in the layers can result in better deep features, deeper layers lead to the learning of less shallow features, which in turn can result in the imprecision and discontinuity of the detail segmentation effect. To avoid the defect and ensure the validity of the extracted features, ASM is introduced as an auxiliary structure to extract the edge information. The ASM is demonstrated in the dashed box in Figure 5. Firstly, to reduce the parameters, an input image is convolved. Subsequently, feature enhancement and edge feature extraction are performed by the CBAM and a dilated convolution, respectively. Finally, the three parts of the feature are fused as the output of the ASM. To better accommodate the extraction needs of the different layers, the rate of the dilated convolution begins from 5 and continuously reduces to 2 with each encoding process. Finally, the results obtained from the ASM and MSM are fused as output. The CEEM can also be given in the following equations:

(1) $$\begin{array}{c} b1=M_{AE}\left(X_{in}\right)=C_{1\times 1}+DC_{3\times 3}\left(C_{1\times 1}\left(X_{in}\right)\right)+C_{CBAM}\left(C_{1\times 1}\right)\text{,}\\ b2=C_{3\times 3}^{n}\left(X_{in}\right)\text{,}\\ X_{out}=b1+b2\text{,} \end{array}$$

where *X*_in_ and *X*_out_ denote the input and output of CEEM, separately, *C*_*k*×*k*_^*n*^ represents the convolution, *k* stands for the size of the convolution kernel, *n* means the amount of powers, and *DC*_*k*×*k*_ suggests the dilated convolution. All convolution operations include batch normalization and ReLU, and *C*_CBAM_ is the CBAM attention.

### 3.3. HSM (Hybrid Subsampled Module)

**Figure 7:**
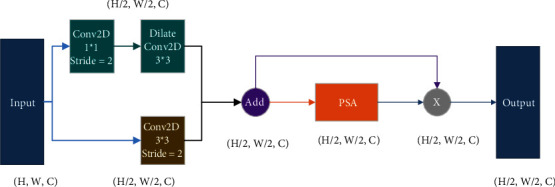
Proposed HSM module.

Currently, pooling operations are used by most semantic segmentation models to reduce the scale of the models and, accordingly, speed up the computations to increase their robustness. However, as can be predicted, the models lose most of their characteristics in the pooling operation. So, to obtain satisfactory results, some scholars have tried engaging different pooling operations in parallel. Get inspired from here, as is shown in Figure 7, and a hybrid subsampled method (HSM) is proposed for AAEHS-Net. Firstly, the input features pass through two branches, one of which reduces the feature parameters by convolution. Subsequently, to obtain more information, the perceptual field is expanded by a dilation convolution. The other branch uses ordinary convolution. To get the results, the features obtained from the two branches are combined. Compared with the pooling operation, the adopted convolution reduces the feature loss. Meanwhile, in order to be adapted to different types of segmentation requirements as well as to obtain richer semantic information, features are fed into a PSA module. PSA applies channel and spatial dimensions to enhance the features. In addition, to learn more initial features and get the final output, the residual structure is used to merge the previous features. This particular process can also be expressed by the following equation:

(2) $$\begin{array}{c} O_{MSM}\left(X\right)=F+W_{PSA}\left(F\right)\\ =f_{3\times 3}\left(X\right)+f_{3\times 3}\left(f_{1\times 1}\left(X\right)\right)+W_{PSA}\left(f_{3\times 3}\left(X\right)+f_{3\times 3}\left(f_{1\times 1}\left(X\right)\right)\right)\text{,} \end{array}$$

where *X* denotes an input, *f*_*k*×*k*_ represents the convolution, *k* stands for the size of the convolution kernel, and *W*_PSA_ means the PSA module.

### 3.4. GAGM (GlobalMaxpool and GlobalAvgpool Module)

**Figure 8:**
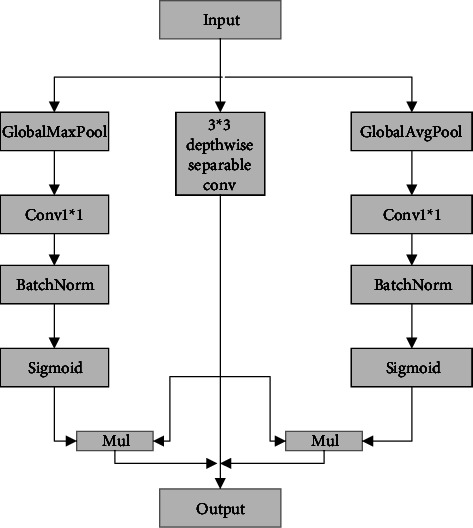
Proposed GAGM.

The attention mechanism is generally used to strengthen the connections between the parts as well as to augment the features. With regards to the pooling operation, while the texture information is extracted by global max pooling, the global information is ignored. Global average pooling, on the other hand, preserves the global information but loses the texture information. However, in order to enhance the input features, CA attention [29] combines pooling operations with an attention mechanism with good results. Inspired by this, GAGM is introduced as a new attention mechanism in this paper, the structure of which is shown in Figure 8. The input features separately run through three branches of global max pooling, depthwise separable convolution, and global average pooling. While depthwise separable convolution is used to obtain more features with the lower computation complexity, global average pooling acquires global features. In addition, global max pooling is applied to focus on the important features. Finally, the results achieved from the three branches are stitched together. The GAGM enriches the feature information extracted by the encoder while effectively combining the global and local information to enhance edge features. The specific formula is as follows:

(3) $$\begin{array}{c} out=\partial \left[f\left(F_{\max}\left(X\right)\right)\right]\times S\left(X\right)+\partial \left[f\left(F_{avg}\left(X\right)\right)\right]\times S\left(X\right)+S\left(X\right)\text{,} \end{array}$$

where *X* denotes input, *S* represents deep separable convolution, *F*_max_ stands for global max pool, *F*_avg_ signifies global average pool, *f* means convolution with BN, *∂* is sigmoid, and *S* suggests the single depthwise separable convolution operation.

### 3.5. Algorithm Step

The specific algorithm flow is shown in Table 2.

## 4. Experiments

First, the datasets used in the experiment are introduced in this section. Next, the details of the implementation and evaluation are provided. Subsequently, the ablation studies carried out to verify the effectiveness of the proposed submodules are presented. Finally, to confirm the advantage of the approach proposed in this study, the presented model is compared with the latest ones.

### 4.1. Datasets

#### 4.1.1. Aerial Drone Image Dataset

**Figure 9:**
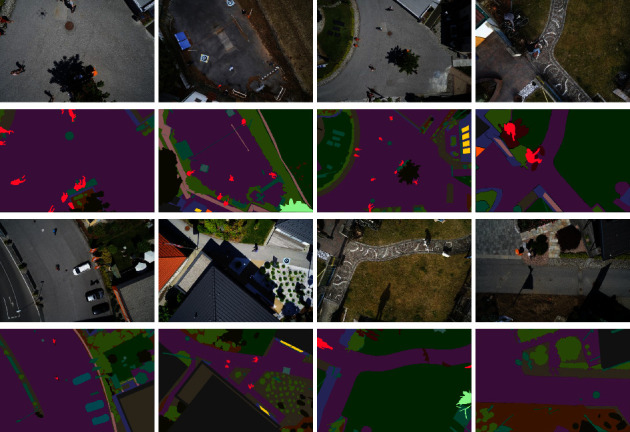
Sample images of the aerial drone image dataset.

The aerial drone image dataset [30] is accessible on Kaggle, which offers 24 categories of datasets. To improve the security of autonomous drone flights and landing sequences, the dataset focuses on the semantic comprehension of urban scenarios. Images depict the picture of more than 20 houses taken from the lowest point (aerial view) at the heights of 5 to 30 meters above the ground. A high resolution camera is used to capture 6000 × 4000 px images. Accordingly, the individual images containing few categories were removed. As the result, a total number of 400 images were obtained. Data enhancement was performed on the data by resizing the images first. Subsequently, the dataset was expanded to 2000 images (1152 × 768 pixels) after applying such data enhancement techniques as random noise addition, horizontal flipping, vertical flipping, and light adjustment and blurring operations. The training, the validation, and the test sets were also divided with a ratio of 80%, 10%, and 10%, respectively. The sample images are shown in Figure 9. Here, the first and third rows are the original images, and the second and fourth rows are the labels.

#### 4.1.2. Massachusetts Roads Dataset

**Figure 10:**
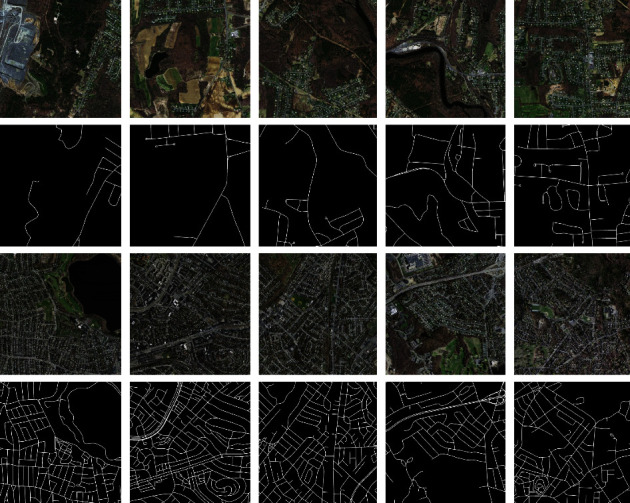
Sample images of the Massachusetts roads dataset.

The Massachusetts roads dataset [31] contains 1171 images (1500 × 1500 pixels). The data were randomly divided into three parts, with a training set of 1108 images as well as a validation set of 14 and a test set of 49 images, respectively. Furthermore, the dataset covers over 2600 km^2^ of various city, countryside, and village areas. The target maps were generated by rasterizing the road centerlines obtained by the open street-map project. Sample images are displayed in Figure 10. In this case, the first and third rows are the original images, and the second and fourth rows are the labels.

#### 4.1.3. Massachusetts Buildings Dataset

**Figure 11:**
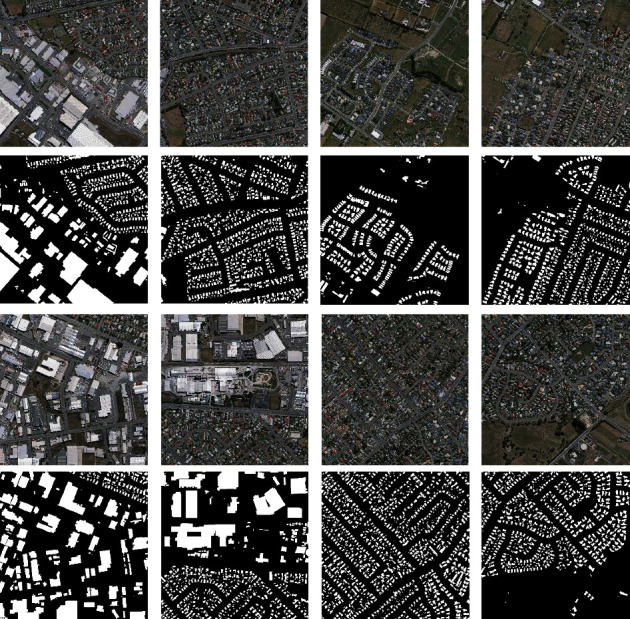
Sample images of the Massachusetts buildings dataset.

The Massachusetts buildings dataset [31] consists of 151 aerial images of the Boston area, with each of the images being 1500 × 1500 pixels for an area of 2.25 square kilometers. The data are split into a training set of 137 images, a test set of 10 images, and a validation set of 4 images. The dataset covers mostly urban and suburban areas and buildings of all sizes, including individual houses and garages, which are included in the labels. In order to train the model adequately, we expand the dataset. Data enhancement is performed on the data by resizing the images first. Subsequently, the dataset was expanded to 1500 images after applying such data enhancement techniques as random noise addition, horizontal flipping, vertical flipping, and light adjustment and blurring operations. The sample images are shown in Figure 11. Here, the first and third rows are the initial pictures and the second and fourth rows are the labels.

### 4.2. Implementation Details and Evaluation

#### 4.2.1. Implementation Settings

To maintain objectivity, the proposed model in this study was implemented using TensorFlow with the same data increments. The model was also trained using a 48 GB Quadro RTX 8000 GPU. In addition, to speed up the process as well as to minimize the loss function, both datasets were evaluated using the Adam optimizer [32]. The initial learning rate obtained was 0.0001. The gradient descent algorithm was implemented as well.  Table 3 shows the remaining hyperparameters.

#### 4.2.2. Evaluation Metrics

To quantitatively evaluate the performance of different models on the Massachusetts roads dataset, four measures, namely, accuracy, MIOU, Dice, and AUC were adopted as the evaluation metrics. However, in the aerial drone image dataset, AUC was not adopted as an evaluation indicator. AUC was defined as the area under the ROC curve. The definitions of accuracy, MIOU, and Dice are given as follows:

(4) $$\begin{array}{c} \text{Accuracy}=\frac{TP+TN}{TP+TN+FP+FN}\text{,}\\ MIOU=\frac{1}{k+1}\overset{k}{\underset{i=0}{\sum }}\frac{TP}{TP+FP+FN}\text{,}\\ Dice=\frac{2TP}{2TP+FP+FN}\text{,} \end{array}$$

where TP, TN, FP, and FN represent true positives, true negatives, false positives, and false negatives, respectively.

### 4.3. Experimental Results

To confirm the effectiveness of AAEHS-Net, extensive experiments were conducted on two image semantic segmentation datasets. In this section, the ablation properties of CEEM, HSM, and GAGM are investigated, compared, and discussed with other semantic segmentation networks.

#### 4.3.1. Ablation Study of CEEM

The impact and significance of the CEEM on the performance of the network are discussed in this section. First, the U-Net is adopted as the baseline model. Next, the feature extraction structure is replaced with the CEEM in the U-Net.  Table 4 provides the specific experimental results of these two methods.

As can be observed in Table 4, U-Net-CEEM obtains the values of 95.47%, 52.11%, and 64.25% for ACC, MIOU, and Dice on the Massachusetts roads dataset, respectively. This is while the values achieved by U-Net were 95.35%, 51.38%, and 63.56%, respectively. Comparison of the two methods reveals an improvement in the values obtained by U-Net-CEEM by 0.12%, 0.72%, and 0.68%, respectively. Therefore, the performance of feature extraction can be argued to be enhanced by the CEEM to a certain extent.

**Figure 12:**
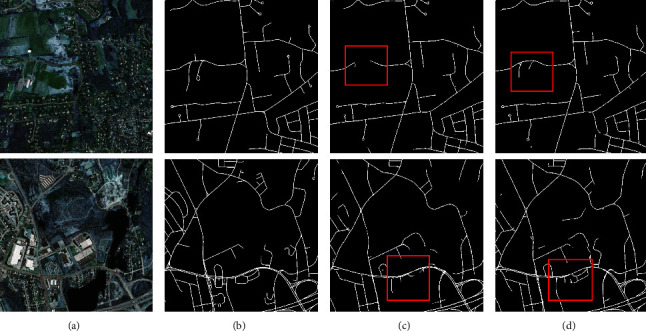
Massachusetts roads CEEM comparison (a) original image, (b) mask image, (c) U-Net, and (d) U-Net-CEEM.

**Figure 13:**
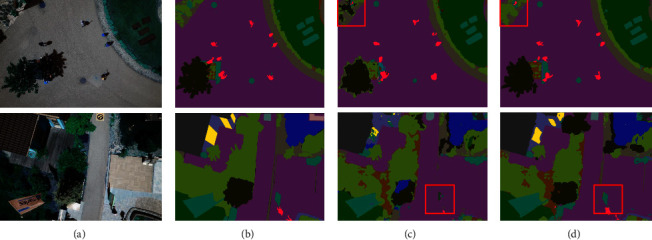
Aerial drone image CEEM comparison (a) original image, (b) mask image, (c) U-Net, and (d) U-Net-CEEM.

To clearly demonstrate the results of the ablation study, the experimental results of two example images are provided in Figures 12 and 13. The red box in the figures shows the better segmentation of the CEEM. This can be argued to be due to the deeper total network layer of the CEEM. As a consequence, more deep information is learned, and the accuracy of the model is improved. Furthermore, more attention is paid to the target regions neglected by the inflated convolution and CBAM attention. Consequently, this is significantly more accurate segmentation of the edge information, which is also well demonstrated on the aerial drone image dataset. To be more exact, ACC, MIOU, and Dice are improved by approximately 0.08%, 0.42%, and 0.29%, with the CEEM, respectively. Hence, the effectiveness of the CEEM is confirmed by the obtained experimental results.

#### 4.3.2. Ablation Study of HSM

The effect of HSM on segmentation performance was also examined. The corresponding segmentation results are obtained on U-Net by sequentially choosing the max pooling operation and using the HSM. The specific experimental results are showed in Table 5.

As shown in Table 5, the highest ACC, MIOU, and Dice are achieved with the HSM on the aerial drone image dataset. The indicators improved by 0.14%, 0.41%, and 0.36% over the baseline network, respectively. HSM also achieves the best performance for all evaluation indicators on the Massachusetts roads dataset.

**Figure 14:**
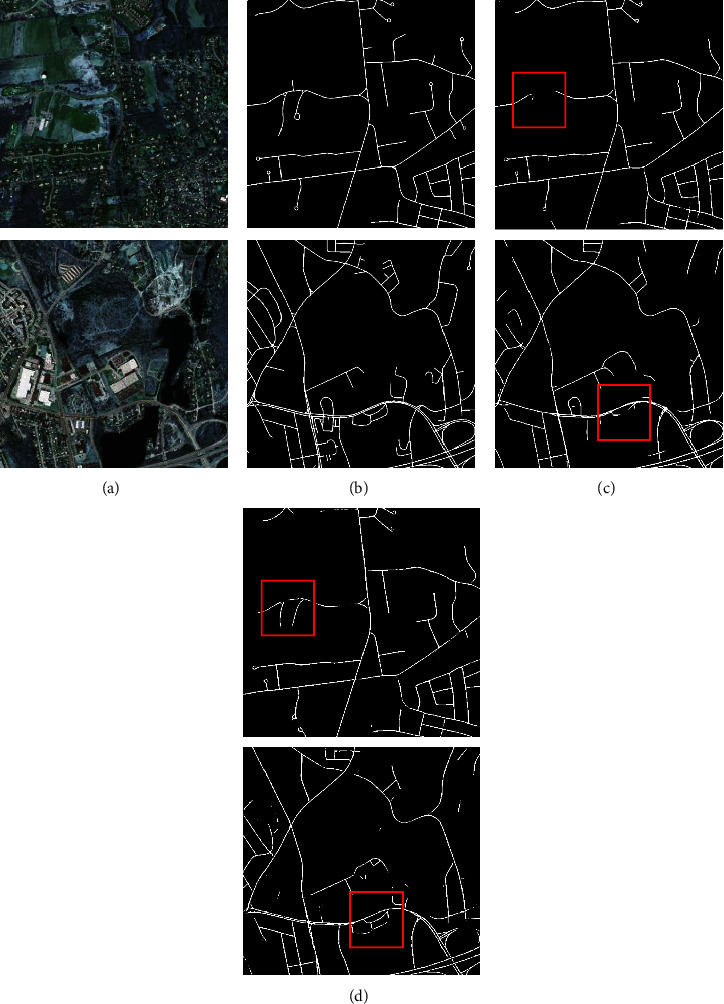
Massachusetts roads HSM comparison (a) original image, (b) mask image, (c) U-Net, and (d) U-Net-HSM.

**Figure 15:**
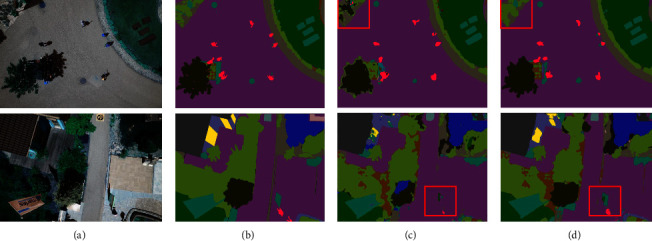
Aerial drone image HSM comparison (a) original image, (b) mask image, (c) U-Net, and (d) U-Net-HSM.

Similarly, to corroborate the experimental results, sample images were given on both datasets, as shown in Figures 14 and 15. The differences between the methods are demonstrated by the red boxes in the diagram. As can be observed, the segmentation network using HSM segmentation is finer and more complete for fine features. This can be argued to be due to the reduction of feature loss during being subsampled in the convolution operation as well as the enhancement of the feature information by PSA attention.

#### 4.3.3. Ablation Study of GAGM

To demonstrate the effectiveness of the GAGM, the segmentation results of the U-Net were also compared with the improved U-Net using the GAGM.  Table 6 shows the corresponding results.

As shown in Table 6, while the values of MIOU and Dice are 51.38% and 63.56% for the U-Net on the Massachusetts roads dataset, respectively, they are 52.43% and 64.29% for the GAGM. This is to say that ACC was improved by 0.28% to 95.63%. Furthermore, ACC, MIOU, and Dice increased by 0.14%, 0.58%, and 0.50% on the aerial drone image dataset with GAGM, respectively.

**Figure 16:**
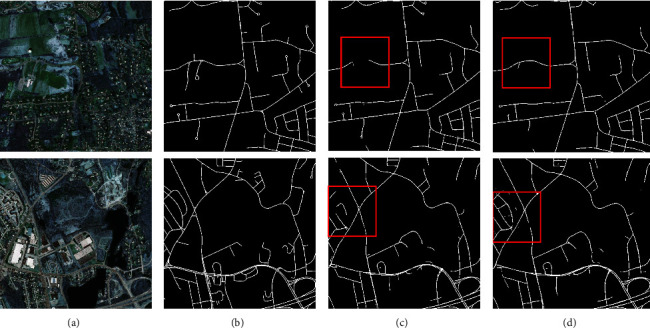
Massachusetts roads GAGM comparison (a) original image, (b) mask image, (c) U-Net, and (d) U-Net-GAGM.

**Figure 17:**
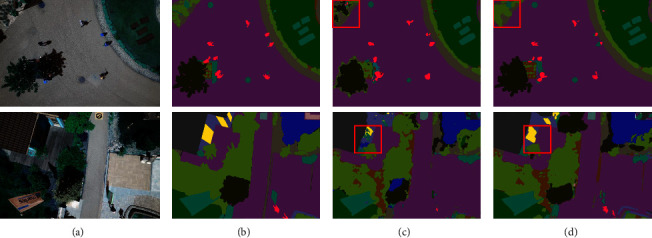
Aerial drone image GAGM comparison (a) original image, (b) mask image, (c) U-Net, and (d) U-Net-GAGM.

This is reinforced by the graphs of the experimental results. As can be observed in Figure 16, the edge contour within the red boxes segmented with GAGM is more continuous. Moreover, as is shown in Figure 17, compared with U-Net, the yellow gates are segmented more accurately with the GAGM, which can be argued to be due to the ability of global average pooling and global max pooling in the GAGM to learn some of the global and important features, respectively. Hence, the improvement of all of the scalar metrics of the network confirms the effectiveness and usefulness of the GAGM.

### 4.4. Comparison of AAEHS-Net with Other Models on Different Datasets

To confirm the validity of AAEHS-Net, the network was also compared with FPN [33], DeepLabv3 [34], NHSU-Net [35], HDA-ResUNet [36], MR-UNet [37], and CE-UNet [38] on different datasets.

#### 4.4.1. Aerial Drone Image Dataset

Table 7 provides the segmentation results of the various networks on the aerial drone image dataset. Compared with other models, the model proposed in this study performs well with regard to the overall segmentation performance. To be more exact, compared with the original U-Net, the MIOU, Dice, and ACC increased by 1.03%, 1.27%, and 1.15% with AAEHS-Net, respectively. In addition compared to FPN and Deeplabv3, Dice improved by 1.74% and 0.9% and achieved the best results in the other two metrics. This is to say that U-Net is not adequate for feature extraction, FPN ignores the enhancement of the features, and Deeplabv3 does not take steps to reduce the feature loss of the down-sampling operation. Hence, their accuracy is lower than that of the algorithm proposed in this study.

Meanwhile, the present model was also compared with the recently proposed algorithmic models. The results revealed the highest level of accuracy, i.e., 90.12% by the model proposed in this study. In addition, the CE-UNet achieved the second highest Dice, with 0.17% below that of our model. Moreover, while MR-UNet uses the coding structure of ResNet34, and consequently, more deep features are able to be extracted; it ignores the shallow information. Although MHSU-Net gains an improved down-sampling operation, the results are still inferior to those of our model. The reason can be argued to be due to the convolution being more efficient, compared with pooling operations. Furthermore, despite all using attention mechanisms, both ACC and MIOU of AAEHS-Net outperform the HAD-ResUNet and CE-UNet, which is due to the effective extraction of edge feature information as well as the reduction of feature loss in the subsampled process in our model.

**Figure 18:**
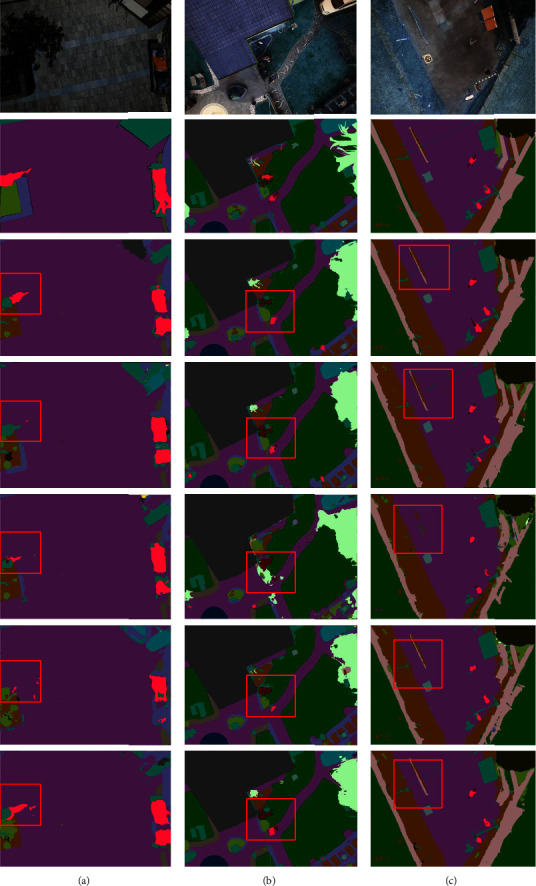
The results of the images tested on the aerial drone image dataset. (a) The original image, (b) the ground truth labeled image, (c) and the predicted labels.

Meanwhile, to clearly demonstrate the segmentation results, they are provided for the sample images of the mainstream algorithms in Figure 18. From the top to the bottom are the original image, the label, MHSU-Net, HAD-ResUNet, MR-UNet, CE-Net, and AAEHS-Net. The parts marked with red circles reveal the best segmentation performance of AAEHS-Net for people in the first and second test images and for nets and wires in the third image. While the MHSU-Net and the HAD-ResUNet networks are able to roughly predict people in the first image and the second test image, respectively, it is only the MR-UNet and CE-Net networks that are incapable of predicting the line in the third image. Hence, it can be concluded that compared with other methods, AAEHS-Net enjoys a stronger segmentation effect.

#### 4.4.2. Massachusetts Roads Dataset

To further evaluate the effectiveness of our approach, experiments on the Massachusetts roads dataset were conducted for the eight methods, the results of which are provided in Table 8.

As can be seen in the table, compared to the various classical networks, AAEHS-Net achieved the best results in all metrics. To be more exact, compared with U-Net, the ACC, Dice, MIOU, and AUC are improved by 0.88%, 1.14%, 1.27%, and 0.89%, respectively. This is an indication of the extraction of sufficient features and retaining the information to the feasible extent by the model proposed in this research. To verify the effectiveness of the network, experimental comparisons were also made with the latest methods. As can be seen in Table 8, this study presented an algorithm which obtained the best results in all evaluation metrics. In particular, compared with HAD-ResUNet, the ACC increased by 0.27% with AAEHS-Net. Moreover, compared with MR-UNet and CE-UNet, MIOU was improved by 0.37% and 0.35%, respectively, and the reason of low accuracy can be argued to be due to the ignorance of the edge features by the networks. Meanwhile, since the features were not well used in down sampling, the Dice of MHSU-Net is 0.69% less than that of our method. Hence, the obtained results of the experiments again corroborate the strong semantic segmentation capability of the model set forth in this study.

**Figure 19:**
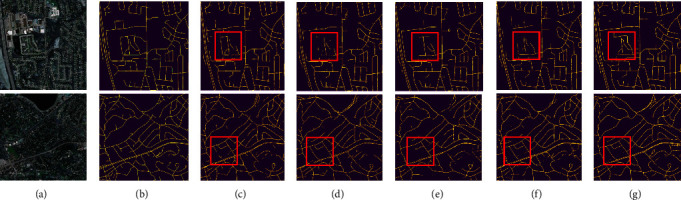
The segmentation results of four currently popular models for two sample images on the Massachusetts roads dataset. (a) Image, (b) mask, (c) MHSU-Net, (d) HAD-ResUNet, (e) MR-UNet, (f) CE-UNet, and (g) AAEHS-Net.

Figure 19 displays the segmentation results of two sample images. As can be observed by the red box, compared with the other methods, the one proposed here integrates the edge information more thoroughly, and accordingly, offering more complete and accurate details. This, in turn, corroborates the effectiveness of AAEHS-Net.

#### 4.4.3. Massachusetts Buildings Dataset

In order to make our method more convincing, eight methods were experimented with on the Massachusetts buildings dataset, and their results are presented in Table 9.

It can be seen from Table 9 that we achieved the best results on the Massachusetts buildings dataset as well. When compared to the baseline network, the accuracy of our proposed network improved by 2.1%, the AUC by 1.59%, and Dice by 2.79%, which fully demonstrates that our proposed individual modules are effective. To further enhance the convincing effect, we are compared with the currently popular algorithms. ACC is improved by 0.87%, Dice by 0.68%, MIOU by 0.33%, and the AUC by 1.11% when compared to the HAD-ResUNet. The reason for this may be that our proposed GAGM is able to enhance global features as well as shallow features, which is not available in the HAD-ResUNet network. Compared to MHSU-Net, ACC is increased by 0.64% and the AUC by 0.96%. This is probably because HSM is subsampled by convolution, which causes less loss compared to pooling operations, and this enhances the features.

**Figure 20:**
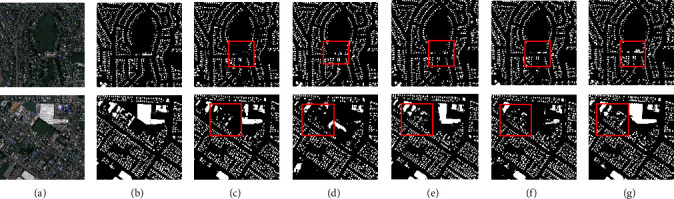
Segmentation results on the Massachusetts buildings dataset. (a) Image, (b) mask, (c) MHSU-Net, (d) HAD-ResUNet, (e) MR-UNet, (f) CE-UNet, and (g) AAEHS-Net.

At the same time, the results of the experiments are plotted to demonstrate the validity of our model.  Figure 20 shows the results of the segmentation on the Massachusetts buildings dataset. From the red box, it is clear that the AAEHS-Net is best for segmentation. This demonstrates the validity of our proposed model.

## 5. Conclusion

AAEHS-Net was proposed as an improved semantic segmentation framework based on U-Net in this research. By using CEEM, AAEHS-Net enhanced the extraction of edge features. Moreover, the replacement of pooling operations with HSM led to the reduction of feature loss. Simultaneously, more global and focused information was obtained through the use of GAGM, which resulted in the enhancement of the features. The network used the above modules to extract more edge features. Meanwhile, the global and focused information pieces of the features were enhanced, and the feature loss was reduced, which ensured the integrity of the contextual information and improved the segmentation accuracy. Compared with the previous works, the experiments on the two datasets confirmed the effectiveness of the proposed model. However, the issue of multiple parameters remained, which will be dealt with through using GAN in our future work.

## Figures and Tables

**Table 1:** Details of CEEM.

Blocks	Input size	Layers	Output size
CEEM1	128 × 128 64 channels	MSM1 ADM1	128 × 128 64 channels
MSM1	128 × 128 64 channels	(Doule conv *k* = 3, *c* = 64, *s* = 1) × 3	128 × 128 64 channels
ASM1	128 × 128 64 channels	Conv2D (*k* = 1, *c* = 64, *s* = 1)	128 × 128 64 channels
		Dilated conv (*k* = 3, *c* = 64, *r* = 4)	
CEEM2	64 × 64 128 channels	MSM2 ADM2	64 × 64 128 channels
MSM2	64 × 64 128 channels	(Doule conv *k* = 3, *c* = 128, *s* = 1) × 4	64 × 64 128 channels
ASM2	64 × 64 128 channels	Conv2D (*k* = 1, *c* = 128, *s* = 1)	64 × 64 128 channels
		Dilated conv (*k* = 3, *c* = 128, *r* = 3)	
CEEM3	32 × 32 256 channels	MSM3 ADM3	32 × 32 256 channels
MSM3	32 × 32 256 channels	(Doule conv *k* = 3, *c* = 256, *s* = 1) × 5	32 × 32 256 channels
ASM3	32 × 32 256 channels	Conv2D (*k* = 1, *c* = 256, *s* = 1)	32 × 32 256 channels
		Dilated conv (*k* = 3, *c* = 256, *r* = 2)	
CEEM4	16 × 16 512 channels	MSM4	16 × 16 512 channels
MSM4	16 × 16 512 channels	(Doule conv *k* = 3, *c* = 512, *s* = 1) × 2	16 × 16 512 channels

**Table 2:** Details of algorithm steps.

Blocks	Input size	Output size
Input	512 × 512 × 3	
Conv2d *c* = 64, *k* = 7, *s* = 2	512 × 512 × 3	256 × 256 × 64
Max pool	256 × 256 × 64	128 × 128 × 64
CEEM1	128 × 128 × 64	128 × 128 × 64
HSM1	128 × 128 × 64	64 × 64 × 64
CEEM2	64 × 64 × 64	64 × 64 × 128
HSM2	64 × 64 × 128	32 × 32 × 128
CEEM3	32 × 32 × 128	32 × 32 × 256
HSM3	32 × 32 × 256	16 × 16 × 256
CEEM4	16 × 16 × 256	16 × 16 × 512
Transpose2d	16 × 16 × 512	32 × 32 × 256
Concat	32 × 32 × 256	32 × 32 × 512
(Conv2d, *c* = 256) *∗* 2	32 × 32 × 512	32 × 32 × 256
Transpose2d	32 × 32 × 256	64 × 64 × 128
Concat	64 × 64 × 128	64 × 64 × 256
(Conv2d, *c* = 128) *∗* 2	64 × 64 × 256	64 × 64 × 128
Transpose2d	64 × 64 × 128	128 × 128 × 64
Concat	128 × 128 × 64	128 × 128 × 128
(Conv2d, *c* = 64) *∗* 2	128 × 128 × 128	128 × 128 × 64
Transpose2d	128 × 128 × 64	256 × 256 × 32
Concat	256 × 256 × 32	256 × 256 × 96
(Conv2d, *c* = 32) *∗* 2	256 × 256 × 96	256 × 256 × 32
Transpose2d *c* = 32	256 × 256 × 32	512 × 512 × 32
Conv2d *c* = 1	512 × 512 × 32	512 × 512 × 1
Output		512 × 512 × 1

**Table 3:** The remaining hyperparameters.

Value	Aerial drone image dataset	Massachusetts roads dataset	Massachusetts buildings dataset
Batch size	8	16	8
Optimizer	Adam	Adam	Adam
Learning strategy	Poly decay	Fixed	Fixed
Beta 1	0.900	0.900	0.900
Beta 2	0.999	0.999	0.999
Epochs	400	300	400

**Table 4:** Results of the ablation study for the CEEM.

	ACC	MIOU	Dice
Methods on the Massachusetts roads dataset			
U-Net	0.9535	0.5138	0.6356
U-Net-CEEM	**0.9547**	**0.5211**	**0.6425**

Methods on the aerial drone image dataset			
U-Net	0.8897	0.5269	0.6870
U-Net-CEEM	**0.8905**	**0.5311**	**0.6899**

The bold value indicates the best value for the item.

**Table 5:** Results of the ablation study for the HSM.

	ACC	MIOU	Dice
Methods on the Massachusetts roads dataset			
U-Net	0.9535	0.5138	0.6356
U-Net-HSM	**0.9558**	**0.5219**	**0.6430**

Methods on the aerial drone image dataset			
U-Net	0.8897	0.5269	0.6870
U-Net-HSM	**0.8911**	**0.5310**	**0.6906**

**Table 6:** Results of the ablation study for the GAGM.

	ACC	MIOU	Dice
Methods on the Massachusetts roads dataset			
U-Net	0.9535	0.5138	0.6356
U-Net-GAGM	**0.9563**	**0.5243**	**0.6429**

Methods on the aerial drone image dataset			
U-Net	0.8897	0.5269	0.6870
U-Net-GAGM	**0.8911**	**0.5327**	**0.6920**

**Table 7:** Results of different models on the aerial drone image dataset.

Method	Accuracy	Dice	MIOU
U-Net	0.8897	0.6870	0.5269
FPN	0.8905	0.6823	0.5252
Deeplabv3	0.8911	0.6907	0.5277
MHSU-Net	0.8933	0.6966	0.5321
HAD-ResUNet	0.8943	0.6953	0.5349
MR-UNet	0.8976	0.6957	0.5347
CE-UNet	0.8985	0.6980	0.5338
Ours	**0.9012**	**0.6997**	**0.5372**

The bold value indicates the best value for the item.

**Table 8:** Results of segmentation on the Massachusetts roads dataset.

Method	Accuracy	Dice	MIOU	AUC
U-Net	0.9535	0.6356	0.5138	0.9082
FPN	0.9517	0.6370	0.5153	0.9056
Deeplabv3	0.9540	0.6326	0.5207	0.9071
MHSU-Net	0.9577	0.6401	0.5231	0.9146
HAD-ResUNet	0.9596	0.6397	0.5242	0.9140
MR-UNet	0.9563	0.6441	0.5228	0.9149
CE-UNet	0.9571	0.6437	0.5230	0.9157
Ours	**0.9623**	**0.6470**	**0.5265**	**0.9171**

The bold value indicates the best value for the item.

**Table 9:** Results of segmentation on the Massachusetts buildings dataset.

Method	Accuracy	Dice	MIOU	AUC
U-Net	0.9305	0.6931	0.6782	0.9524
MHSU-Net	0.9451	0.7155	0.6781	0.9587
HAD-ResUNet	0.9428	0.7142	0.6802	0.9574
MR-UNet	0.9462	0.7179	0.6798	0.9620
CE-UNet	0.9490	0.7137	0.6811	0.9598
Ours	**0.9515**	**0.7210**	**0.6835**	**0.9683**

The bold value indicates the best value for the item.

## Data Availability

The data used to find the study can be available upon request to the corresponding author.
